# Physiology of Cardiac Arrest with del Nido Cardioplegia

**DOI:** 10.31480/2330-4871/199

**Published:** 2025-02-10

**Authors:** McKenna Longacre, Juan C. Ibla, Koichi Yuki

**Affiliations:** 1 Division of Cardiac Anesthesia, Department of Anesthesiology, Critical Care and Pain Medicine, Boston Children’s Hospital, Boston, MA, 02115, USA; 2 Department of Anaesthesia, Harvard Medical School, Boston, MA, 02115, USA

**Keywords:** del Nido cardioplegia, Intracellular calcium, Anaerobic metabolism

## Abstract

Del Nido cardioplegia is widely used in current pediatric cardiac surgery practice in North America and its application is also being extended to adult cardiac surgical procedures. Here we will review the physiology of del Nido cardioplegia based on our personal discussion with Dr. del Nido and the published literature.

## Introduction

The implementation of cardioplegia has increased the safety of cardiac surgery by reducing ischemic injury to myocytes during cardiopulmonary bypass (myocardial protection), and has increasing the scope of surgery, enabling a still, bloodless field. Several cardioplegia solutions are available in the US and abroad, with significant variability with respect to crystalloid bases, temperature, intracellular versus extracellular delivery, addition of blood, electrolyte composition, osmolality, and addition of sodium channel blockers, etc. Of these, del Nido cardioplegia, developed at the University of Pittsburg in the early 1990s, is the only commercially available formulation specifically for the neonatal myocardium (Table 1).

## Physiologic Principals of del Nido Cardioplegia

There are several important differences between neonatal and adult myocardium. While some of these nuances are still debated [1], the neonatal myocardium is likely less tolerant of ischemia, and more sensitive to an increase in intracellular calcium, particularly during reperfusion [1–4]. To address this increased vulnerability, del Nido cardioplegia was designed to provide additional myocardial protection by preventing intracellular sodium -and in turn Ca^2+^- accumulation in the cells and to support anaerobic metabolism to maintain intracellular high energy phosphate reserves during ischemia.

### Preventing accumulation of intracellular calcium during ischemia

Electrical arrest of the heart is achieved via depolarization. This is generally achieved by the delivery of high dose potassium, often in conjunction with hypothermia. This strategy has the advantage of providing a quick arrest. However, depolarization also leads to intracellular accumulation of sodium and calcium, which has been associated with poor myocardial recovery following reperfusion [5,6].

Myocardial function is driven by the influx and efflux of Ca^2+^, with influx inducing actin and myosin interactions that result in contraction. The influx of Na^2+^- and in turn Ca^2+^- can induce energy-consuming contracture, both promoting ischemia and irreversible mitochondrial injury [1]. Addition of a sodium channel blocker can therefore decrease harmful intracellular accumulation of Ca^2+^. In del Nido Cardioplegia, lidocaine is used for this purpose due to its longer half-life (as compared to procaine in St Thomas cardioplegia), and yet relatively quick onset of action. This longer half-life has the added advantage of orchestrating a slower resumption of electrical activity after reperfusion, which allows recovery of mitochondria and ATP before intracellular Ca^2+^ pumps resume function. Magnesium cations are also added to compete with Ca^2+^ influx [5,7].

### Optimizing aerobic and anaerobic metabolism

The goal of cardioplegia is to meet the ongoing energy needs of the arrested heart by decreasing the overall energy requirement and optimizing metabolism of the nonperfused heart. A significant innovation of del Nido solution was the addition of hyper-oxygenated blood (comprising 20% of the total solution). Functionally, the cardioplegia solution is “made” once the patient is on cardiopulmonary bypass by mixing 4 parts cardioplegia solution with 1 part blood which has been fully oxygenated by the cardiopulmonary bypass circuit (often with pO_2_ of > 500 mmHg). This provides a limited reservoir of O_2_ for aerobic metabolism in the coronary arteries during arrest. Additionally, red blood cells provide a potent buffer via intracellular carbonic anhydrase (${\text{HCO}}_{2}^{-}\to {\text{CO}}_{2}$ and H_2_O), and scavenge free radicals including superoxide anion, hydrogen peroxide, and hydroxyl [1].

Another innovation of del Nido cardioplegia was recognition that exogenous glucose is toxic during periods of ischemia [8]. This is likely due to the fact that glucose transporters (GLUT-4) also carry sodium into the cell, and thus contribute to the accumulation of intracellular Ca^2+^ as discussed above. This relationship between exogenous glucose and intracellular calcium influx likely explains the observation that blood cardioplegia solutions based on 5% dextrose require multi-dosing to maintain some aerobic metabolism and hyperpolarization.

As such, del Nido cardioplegia relies on endogenous glycogen breakdown for anaerobic metabolism, which is ample in healthy myocardium. This anaerobic metabolism is further supported by the addition of red blood cells and added bicarbonate to neutralize free hydrogen ions, which are otherwise inhibitory to anaerobic metabolism. Increased anaerobic lactate production is likely particularly advantageous during early reperfusion, when energy requirements rapidly increase.

### Other components of del nido cardioplegia

Del Nido cardioplegia solution also contains mannitol for free radical scavenging, and to reduce myocardial cell swelling [2,9–12]. This may be particularly advantageous in long bypass cases with large volume transfusion, such as many complex neonatal cases.

At Boston Children’s hospital, Plasma-Lyte A is used as the crystalloid base. However, no difference base been found between Plasma-Lyte A as base and other crystalloid solutions with a physiologic sodium (Lactated Ringers) [13,14]. Plasma-Lyte A has the possible advantage of containing no calcium, such that the only source of calcium is from the added red blood cells. Interestingly, cardioplegia solutions with trace calcium have historically out-performed acalcemic preparations (the calcium paradox) (Table 2) [15].

At our institution, del Nido cardioplegia is passed through cooling coils on ice prior to delivery, yielding a delivery temperature 8–12 °C [1]. This hypothermia decreases metabolic demand of the myocardium, while also facilitating arrest.

### Technical considerations

The patent for del Nido cardioplegia expired in 2003 and as such, the solution can now be made in-house or purchased from vendors (Table 3) [16]. This has contributed to its increased use in the US and abroad.

At our institution, it is generally administered as a single 20-mL/kg dose, with a maximum arresting dose of 1 L for patients larger than 50 kg [1]. Additional cardioplegia doses are given under special circumstances, including hypertrophy, aortic insufficiency, and surgeon preference [1]. In the North America survey published in 2013, the average redosing interval was ~45 min [17], though in our experience it can be dosed less frequently than this without myocardial injury. While there is no data available, it may be advantageous to limit the mechanical trauma associated with the delivery of cardioplegia, particularly to neonatal coronaries. At Boston Children’s, we utilize a modified delivery system with 1 cc of dead space to ensure a full dose of cardioplegia is delivered to the patient, which may further contribute to infrequent need to redoes [1].

### Comparative efficacy

There is an emerging body of literature comparing the efficacy of del Nido cardioplegia versus other conventional solutions in adult patients [18–24]. Most studies include relatively short, low complexity cases [25–27]. For example, Kim, et al. at the Cleveland Clinic retrospectively reviewed the use of de Nido to Buckberg blood cardioplegia in adults undergoing aortic or mitral valve repair. They found no difference with respect to safety, and suggested possible advantages including fewer interruptions to re-dose, easier glycemic control, and reduced surgical time [25]. However, at present there are no prospective clinical trials of del Nido cardioplegia in pediatric or adult populations. Give the increasing use in adults, there is need for a comprehensive assessment of myocardial protection, including myocardial enzymes, echocardiogram data, cardiac output, and overall outcomes, particularly in complex adult cases [26].

## Conclusion

Here we reviewed the pertinent physiology of del Nido cardioplegia. While its efficacy is widely recognized, further study is needed for a comprehensive assessment of del Nido cardioplegia versus other cardioplegia solutions in both pediatric and adult patient populations.

## Figures and Tables

**Table 1:** Del Nido cardioplegia solution composition.

1 L Plasma-Lyte A base solution (140 mEq/L sodium, 5 mEq/L potassium, 3 mEq/L magnesium, 98 mEq/L chloride, 27 mEq/L acetate, and 23 mEq/L gluconate; Baxter Healthcare Corporation, Deerfield, IL)
Mannitol 20%, 16.3 mL
Magnesium sulfate 50%, 4 mL
Sodium bicarbonate 8.4%, 13 mL
Potassium chloride (2 mEq/mL), 13 mL
Lidocaine 1%, 13 mL

**Table 2:** Comparison of acalcemic with trace calcium containing cardioplegia.

Acalcemic cardioplegia	Trace calcium containing cardioplegia
NaCl 27 mmol/L	NaCl 27 mmol/L
KCl 30 mmol/L	KCl 30 mmol/L
MgSO_4_ 3 mmol/L	MgSO_4_ 3 mmol/L
Glucose 83 mmol/L	Glucose 83 mmol/L
L-Histidine 195 mmol/L	L-Histidine 195 mmol/L
THAM 4	CaCl_2_ 70 μmol/L
pH 7.8	THAM 4
	pH 7.8

**Table 3:** Methods to obtain del Nido cardioplegia at select US pediatric hospitals.

Institution	Methods to Obtain del Nido Cardioplegia	
	Vendor	Made in House
Toronto Sick Kids (Toronto)		X
Boston Children’s Hospital (Boston)	X	X
Children Hospital of Philadelphia (Philadelphia)	X	
Nicklaus Children’s Hospital (Miami)	X	X
Emory Children’s Center (Atlanta)		X
Lurie Children’s (Chicago)	x	
UCSF Benioff Children’s Hospital (San Francisco)	x	
Lucile Packard Children’s Hospital (Palo Alto)	x	
Seattle Children’s Hospital (Seattle)	x	X
Texas Children’s Hospital (Houston)		X

Based on personal communication in Sep 2024
